# Correlative Electrochemical Microscopy of Li‐Ion (De)intercalation at a Series of Individual LiMn_2_O_4_ Particles

**DOI:** 10.1002/anie.201814505

**Published:** 2019-02-21

**Authors:** Binglin Tao, Lewis C. Yule, Enrico Daviddi, Cameron L. Bentley, Patrick R. Unwin

**Affiliations:** ^1^ Department of Chemistry University of Warwick Coventry CV4 7AL UK

**Keywords:** batteries, electrochemistry, LiMn_2_O_4_, Scanning electrochemical microscopy, single-particle analysis

## Abstract

The redox activity (Li‐ion intercalation/deintercalation) of a series of individual LiMn_2_O_4_ particles of known geometry and (nano)structure, within an array, is determined using a correlative electrochemical microscopy strategy. Cyclic voltammetry (current–voltage curve, *I*–*E*) and galvanostatic charge/discharge (voltage–time curve, *E*–*t*) are applied at the single particle level, using scanning electrochemical cell microscopy (SECCM), together with co‐location scanning electron microscopy that enables the corresponding particle size, morphology, crystallinity, and other factors to be visualized. This study identifies a wide spectrum of activity of nominally similar particles and highlights how subtle changes in particle form can greatly impact electrochemical properties. SECCM is well‐suited for assessing single particles and constitutes a combinatorial method that will enable the rational design and optimization of battery electrode materials.

As a promising Li‐ion battery cathode material in both aqueous and organic electrolytes, spinel LiMn_2_O_4_ has attracted much attention in recent years owing to its large theoretical capacity, high abundance, and nontoxicity,^1^ although a number of problems remain to be resolved.^2^ As with much research in electrochemistry, macroscale electrochemical measurements have mainly been used to study battery materials, which for complex composite electrodes include contributions from the conductive agent, adhesive, and the active material.^3^ Local structure–activity relationships for battery materials are still somewhat unexplored in regards to individual active particles or particle agglomerates.^4^ Indeed, the variation in activity among particles, and the relation to particle topology and structure, has largely remained elusive. This paper addresses this issue head on, through the use of a strategy that enables the measurement and direct comparison of the structure and electrochemical activity of individual particles.

In order to rationally design battery electrodes, and electroactive materials in general, strategies that enable the direct correlation between the local redox activity and electrode structure are highly valuable.^5^ In addition to some optical approaches (e.g., plasmonic imaging^6^), emerging in situ scanning electrochemical probe microscopy (SEPM)^7^ techniques also promise to provide insight into the structural factors controlling the electrochemical behavior of battery electrode materials. Within the SEPM family, scanning electrochemical microscopy (SECM) has been the most widely used in Li‐ion battery research, especially for probing the electrically insulating solid electrolyte interface (SEI), although mainly on the scale of tens of microns.^8^ Scanning ion conductance microscopy (SICM) offers a much higher spatial resolution and has been used to visualize ion‐flux spatial heterogeneities in tin and silicon anodes in Li‐ion batteries.^9^ It is worth noting that both SECM and SICM collect electrochemical information about an electrode substrate by monitoring the spatially dependent concentrations/fluxes of the reactant, product, or intermediates at a scanning electrode tip.

In contrast, in scanning electrochemical cell microscopy (SECCM), electrochemistry is probed directly and locally at a substrate electrode, with a spatial resolution defined by the area of meniscus contact, and with the possibility of synchronous co‐location topographical mapping.5a, 10 In the context of battery research, this technique has previously been used to electrochemically interrogate thin films of (insulating) Li_2_O_2_,^11^ as well as small populations of LiFePO_4_ particles (ca. 10 particles).^4^, ^12^ In this study, SECCM has been deployed in a single‐channel nanopipette configuration to investigate the electrochemical behaviour of individual LiMn_2_O_4_ particles within an ensemble, which were visualized, post‐experiment, by co‐located SEM. Experimental details are available in the Supporting Information, Section S1.

SECCM was deployed in hopping mode,5a, 13 as shown schematically in Figure 1 a (labelled in the Supporting Information, Section S2, Figure S1). In this configuration, a nanopipette probe, containing 1.0 m aqueous LiCl as the electrolyte and a AgCl‐coated Ag wire as a quasi‐reference counter electrode (QRCE), was approached to the substrate (working electrode) surface to make meniscus contact at a series of predefined locations in a grid (Figure S2). At each landing, local electrochemical measurements (*I*–*E* or *E*–*t*) were made within the confined area defined by the meniscus cell (the probe itself did not make physical contact with the surface). The substrate was prepared by drop casting spinel LiMn_2_O_4_ particles onto glassy carbon (GC; Figure S3).

**Figure 1:**
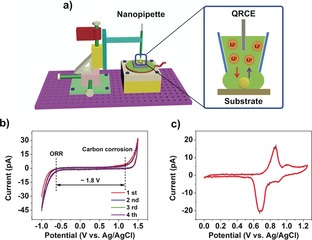
a) Schematic showing the sub‐microscale electrochemical measurements performed on single LiMn_2_O_4_ particles using SECCM. On the right is an enlarged diagram of the probe‐particle‐support interface at a single pixel of a scanning experiment, in which an individual LiMn_2_O_4_ particle is fully encapsulated by the meniscus cell. b) Four SECCM CVs obtained at the GC support and c) a typical CV obtained from a single LiMn_2_O_4_ particle. Experiments performed in 1 m LiCl, with a 500 nm diameter probe, at a scan rate (*ν*) of 1 V s^−1^.

To explore the Li^+^ storage mechanism at individual LiMn_2_O_4_ particles, as well as visualize the variation in activity within an active ensemble, spatially resolved cyclic voltammetry was performed on the as‐prepared LiMn_2_O_4_/GC electrode. Starting at 0 V vs. Ag/AgCl, the potential was swept between 0 to 1.25 V at a rate of 1 V s^−1^. As shown in Figure 1 b, a relatively featureless cyclic voltammogram (CV) was obtained on the GC support, with processes encountered at extreme anodic and cathodic potentials attributable to carbon corrosion^14^ and the oxygen reduction reaction (ORR),^15^ respectively. Thus, the electrochemical stability window of GC was estimated to be approximately 1.8 V under these conditions.

Figure 1 c depicts a representative CV obtained at a single LiMn_2_O_4_ particle, encapsulated by the meniscus (droplet) cell. Li^+^ (de)intercalation chemistry at LiMn_2_O_4_ can be expressed by Eq. (1):

(1) $$\mathrm{LiMn}{}_{2}O{}_{4}\vec{\leftarrow }x\;\mathrm{Li}{}^{+}\;+x\;e{}^{-}\;+\;\mathrm{Li}{}_{1-x}\mathrm{Mn}{}_{2}O{}_{4}$$

where typically 0<*x*<1.^16^ During the charging process [Eq. (1), forward], Li^+^ is extracted from the structural framework of LiMn_2_O_4_, coinciding with the oxidation of Mn^III^ to Mn^IV^. This corresponds to the sweep in the positive direction, in which two redox peaks located at 0.89 and 1.01 V vs. Ag/AgCl (1.0 m LiCl) can be assigned to Li^+^ extraction from tetrahedral lattice sites in the presence and absence of the Li–Li interaction, respectively.^16^ The reverse processes [Eq. (1), reverse] occurred during discharge, with the two peaks at 0.69 and 0.89 V in the negative sweep corresponding to the two different Li^+^ insertion processes. In addition, no undesirable side (parasitic) reactions were observed at high potentials, demonstrating that the oxygen evolution reaction does not occur on LiMn_2_O_4_ in this potential range.^17^ It is interesting to note that this scan rate (*ν*) is 2–4 orders of magnitude larger than that employed in bulk electrochemical experiments with the same material (*ν*=0.1–10 mV s^−1^),^18^ and yet the (de)intercalation processes are facile. This indicates that in the traditional composite electrode configuration, the achievable (de)intercalation rates are largely governed by the rate of electron transfer between the auxiliary elements (e.g., binder and carbon black) and electroactive components (see below). Note that the low currents passed during measurement in SECCM makes it relatively immune to resistance arising from the sample itself (e.g., low intrinsic conductivity or contact resistance), making this technique ideal for the study of a diverse range of (semi)conductive materials.5a, 19

Individual LiMn_2_O_4_ particles within the ensemble were probed in an automated fashion by performing a hopping mode SECCM scan in the voltammetric mode, in which each hop corresponds to an independent, spatially resolved CV experiment.5a, 13 The hopping distance (i.e., distance between each landing/pixel) was 1.5 μm, which ensured each measurement spot was independent of the last. An SEM image of the probed area, post‐scan, is shown in Figure 2 a (also shown enlarged in Figure S4). Evidently, the probed area is predominantly GC (individual droplet footprints are visible in the scan area), with a collection of LiMn_2_O_4_ particles scattered throughout. A comparison with the SECCM topographical (*z*‐height) map in Figure 2 b, revealed 18 pixels with elevated topography, each corresponding to an isolated LiMn_2_O_4_ particle or agglomerate (see below). The co‐location of the particles (Figure 2 a) and the higher points in the topography map (Figure 2 b) gives us confidence that the SECCM technique can be used to identify particles in situ.

**Figure 2:**
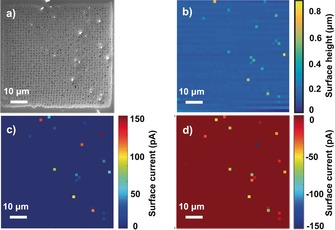
SECCM‐CV measurements of individual and aggregated LiMn_2_O_4_ particles supported on GC. a) SEM image and b) topography (*z*‐height) of the corresponding scanning area. Surface current maps obtained at c) 1.0 V (forward sweep) and d) 0.6 V (reverse sweep) during the anodic and cathodic scan, respectively.

A spatially resolved CV‐SECCM movie (current maps as a function of potential) obtained on the LiMn_2_O_4_/GC ensemble electrode (60×60 μm^2^, 40×40 pixels) is shown in the Supporting Information, Section S3 and Movie S1. The magnitude of the anodic and cathodic currents (i.e., peak current) obtained at each individual active pixel is comparable throughout, signifying that Li^+^ (de)intercalation is relatively reversible (see below). Figure 2 c,d depicts two frames from the movie, taken from the anodic (forward) and cathodic (reverse) sweeps at potentials of 1.0 V and 0.6 V, respectively. Through correlation of the activity maps with the SEM image of the scan area (Figure 2 a) and surface topography map (Figure 2 b), it is obvious that the individual LiMn_2_O_4_ particles exhibit elevated currents compared to the relatively inert GC support. It should be noted that while a CV‐scan hopping protocol was employed above, chronoamperometric (current–time curve, *I*–*t*) waveforms can also be applied if only a single potential is of interest, as shown in Figure S5.

The individual LiMn_2_O_4_ particles (including primary particles and agglomerated secondary particles) exhibit very different current magnitudes in Figure 2 c,d, indicative of heterogeneous size and activity within the ensemble. Indeed, by extracting the individual CVs from each active pixel, as shown in Figure S6, it is clear that each particle/agglomerate presents a unique *I*–*E* profile, attributable to its physical heterogeneities (e.g., particle size, composition, crystallinity, or orientation), as demonstrated by the corresponding high‐resolution SEM images in Figure S7. It is worth reemphasizing, the variation in *I*–*E* characteristics (“activity”) among superficially similar particles (or agglomerates) is completely invisible in macroscopic (bulk) measurements, which reflect the average response of the ensemble (see below). As the probed area (indicated by the individual droplet footprints) is only a little bit larger than the tip diameter (500 nm, Figure S8), some LiMn_2_O_4_ agglomerates cannot be fully encapsulated by the SECCM meniscus. In order to treat the data semi‐quantitatively (i.e., the active particle surface area is known, see below), the meniscus cell should totally encapsulate the particle during measurement, as shown schematically in Figure 1 a. Thus, multiple scans were performed on different areas of the LiMn_2_O_4_/GC ensemble and only pixels in which particles were small (or sparse) enough to be fully encapsulated by the meniscus were selected for comparison and quantitative analysis, as depicted in Figure 3. A further indication of the validity of this approach is that the overall peak currents fall within a fairly narrow range of circa 30–70 pA, notwithstanding some variation in the peak potentials and overall CV morphology. Note that the size of the nanopipette probe could easily be tailored to accommodate encapsulation of larger particles, or smaller particle‐to‐particle separations.

**Figure 3:**
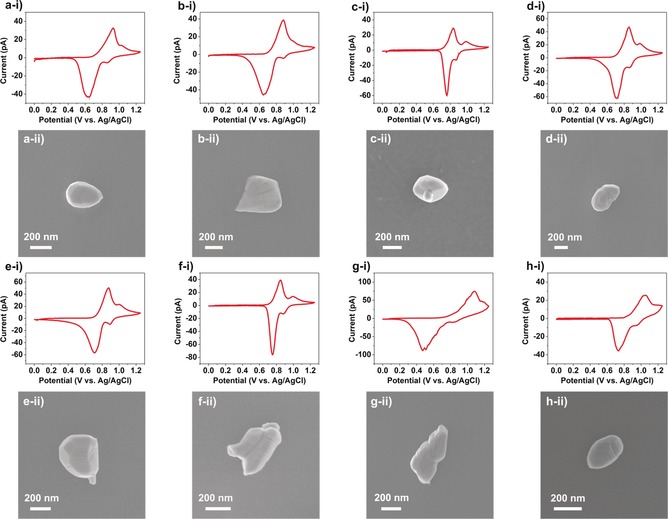
a–h) CVs (i) and corresponding SEM images (ii) from individual LiMn_2_O_4_ particles supported on GC. The CV measurements (*ν*=1 V s^−1^) were obtained by local ensemble measurements with SECCM, with a 500 nm diameter probe filled with 1 m LiCl.

The magnitude of the current measured at each pixel is governed by the size (i.e., the exposed surface area) of the LiMn_2_O_4_ particle, while the position and shape of the anodic and cathodic peaks, indicative of the Li^+^ (de)intercalation mechanism and kinetics, is governed by the particular properties (i.e., composition, crystallinity, and orientation) of the particle. It is important to note that the electrochemical behaviour of individual LiMn_2_O_4_ particles is highly heterogeneous, with the voltammetric peak morphology (position, separation, and width) varying considerably throughout the ensemble. Some particles, such as particles (g) and (h), exhibit very sluggish kinetics (i.e., large peak‐to‐peak separations), which is not desirable for the application of this material as an active battery material. By comparison, particle (c), which appears to be comprised of small crystallographic facets, exhibits fast kinetics, making it the ideal structure that should be pursued through the application of novel design principles. To further illustrate this point, detailed comparisons of the electrochemical properties (voltammetric peak potential and current, total charge and cathodic‐to‐anodic charge ratio) of each individual particle in Figure 3 are summarized in Table S1. A particularly interesting observation is that the cathodic‐to‐anodic charge ratio (calculated by dividing the total cathodic charge by the total anodic charge) is higher than 100 % for all particles, which is ascribed to the Jahn–Teller effect.^20^ In brief, a fraction of the Mn^3+^ is further reduced to Mn^2+^ during the reverse scan (Li^+^ intercalation process), which subsequently undergoes dissolution into the electrolyte. Thus, the material is over‐reduced, resulting in enhanced cathodic charge and an apparent cathodic‐to‐anodic charge ratio greater than 100 % during cycling. As the CV measurement only probes the near‐surface processes (i.e., only 10–30 % of the total capacity can be used), this phenomenon can carry on for multiple cycles without apparent capacity loss (Figure S9). Besides this, the voltammetric peak‐to‐peak separation (Δ*E*_p_) is observed to decrease during the multiple voltammetric cycling, indicating that the (de)intercalation processes become kinetically more facile at the single particle level.

To further clarify the relationship between single particle and conventional macroscale electrochemistry, voltammetry was performed on a composite (i.e., material, binder, and conductive additive) LiMn_2_O_4_ electrode (Figure S10). Note that in bulk only a fraction of the total capacity is accessed (e.g., 23 % at 5 mV s^−1^) and the cathodic‐to‐anodic charge ratio is greater than 100 %, in agreement with the single‐particle measurements above, as well as previous reports.^21^ Viewing these results alongside those from SECCM (Figure 3), it is very clear that the bulk electrochemical response “washes out” the unique properties of each individual LiMn_2_O_4_ particle. This contrasts with the SECCM measurements, which reveals the heterogeneity of activity at the single particle level. To illustrate this point further, the 8 CVs in Figure 3 were averaged (Figure S11) to produce a curve that superficially resembles (i.e., two anodic peaks observed at 0.8 and 1.0 V) the bulk ensemble response. It should also be noted that the bulk composite electrode response can be reproduced in the SECCM configuration at low *ν* using large, micrometric probes (8 and 50 μm in diameter, Figure S8), in which a collection of LiMn_2_O_4_ particles are probed during each experiment (Figure S12). This demonstrates that the diversity of responses observed in Figure 3 must arise from intrinsic differences between the LiMn_2_O_4_ particles, rather than being an artefact of the SECCM configuration or the high *ν* used, again underscoring the importance of kinetic effects in Li^+^ (de)intercalation reactions.

To complete this study and highlight further the versatility of the SECCM approach, spatially‐resolved galvanostatic charge–discharge measurements were performed at the single particle level, with an applied current of ±5 pA for 1 s at each measurement point. Spatially resolved, potential–time snapshots (maps) obtained at different times and current polarities are presented in Figure S13 a–d. Again, by comparing the maps with the corresponding SEM image in Figure S13 e, it is clear that different particles present different charge/discharge potentials, attributed to unique structural characteristics (i.e., size and morphology). Figure S13 f shows a representative *E*–*t* curve (galvanostatic charge/discharge profile) extracted from a single LiMn_2_O_4_ particle, in which the charge/discharge processes occur at a potential of circa 0.75 V vs. Ag/AgCl, which is consistent with the peak position in the CVs shown in Figure 3. In contrast, at GC, the measured potential changes rapidly (non‐faradaic or capacitive charging current) before reaching the electrochemical window limits highlighted in Figure 1 b, as expected for an ideal polarizable electrode system.

Figure 4 depicts the galvanostatic charge–discharge measurements performed on individual LiMn_2_O_4_ particles (agglomerates) that again, are small enough to be fully encapsulated by the SECCM meniscus (electrochemical cell). In line with the CV results above, each particle presents a unique *E*–*t* profile, with different charge/discharge potentials and ohmic (*IR*, where *R* is resistance) drops (i.e., the potential difference between the charge/discharge plateau), as summarized in Table 1. Again, it needs to be reiterated that the heterogeneity in activity (*E*–*t* profiles in Figure 4 or CVs in Figure 3) among superficially similar LiMn_2_O_4_ particles or agglomerates is a largely unexplored phenomenon that is obscured in traditional macroscopic measurements on composite electrodes. It should also be noted that the *IR* drop values are very low, especially considering the extremely high charge/discharge rates implemented in this study (e.g., the *IR* drop was only ca. 20 mV at a C‐rate of 279 C for particle b in Figure 4). This value is among the highest C‐rates reported in the literature, with high rate performance Zn (up to 50 C) and Al (up to 500 C) ion battery electrodes being reported before.^22^ As alluded to above, this indicates that in the traditional composite electrode configuration, *IR* drop (and hence rate‐performance limitation) is largely governed by the rate of electron transfer between the auxiliary elements (e.g., binder and carbon black) and electroactive component(s), rather than Li^+^ (de)intercalation into the individual LiMn_2_O_4_ particles. Thus, there remains great potential to further improve the rate capability in battery electrochemistry by new strategies to wire active particles or by improving the electrode preparation method to enhance the charge transfer kinetics (see above).^23^ It needs to be reiterated that the timescale of these localized *E*–*t* experiments is orders‐of‐magnitude faster than that usually encountered in bulk electrochemical measurements (i.e., 0.1 to 10 C rates), which is explored in detail in Section S4.

**Figure 4:**
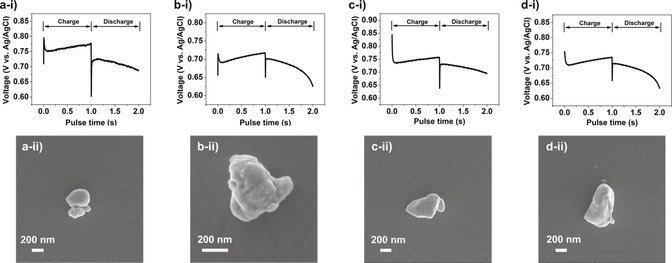
a–d) Galvanostatic charge–discharge curves (i) and corresponding SEM images (ii) from individual LiMn_2_O_4_ particles supported on GC. The charge–discharge measurements (*I*=±5 pA) were obtained by local ensemble measurements with SECCM, with a 500 nm diameter probe filled with 1 m LiCl.

**Table 1:** Physical and electrochemical characteristics of each particle investigated by galvanostatic charge/discharge.

Particle^[a]^	a	b	c	d
*E*_charge_ [V]	0.763	0.705	0.747	0.723
*E*_discharge_ [V]	0.713	0.685	0.719	0.698
Volume^[b]^ [×10^−14^ cm^3^]	3.3	3.0	7.7	12.9
Capacity^[c]^ [pC]	73	64	168	282
C rate	247	279	107	64
*IR* drop [mV]	50	20	28	25

[a] Particle labels correspond to those in Figure 4. [b] The volume of each particle was estimated based on the height (estimated from *z*‐height topography), width, and length (estimated from SEM image) by assuming the particle is an ellipsoid (*V*=4/3π*a* *b* *c*). [c] The capacity calculation process can be found in the Supporting Information, Section S4.

In summary, using a mobile meniscus cell in the SECCM configuration, we have been able to probe and compare the electrochemical activities of individual particles within an ensemble. This direct and local probe method has enabled characteristic features to be targeted and analysed precisely through a correlative approach with ex situ SEM. Specifically, in this work LiMn_2_O_4_, a promising Li‐ion battery cathode material, has been revealed to possess significantly heterogeneous electrochemical behaviour [i.e., Li^+^ (de)intercalation processes] at the single particle level, attributable to differences between particle size, composition, crystallinity, and orientation. In addition, the variation in electrochemical activity revealed by these sub‐microscale (single particle) measurements has allowed us to rationalize the macroscopic bulk electrochemical response of complex composite battery electrodes.

In the past few years, a number of in situ/in operando analysis tools have been established for the exploration of complex redox processes in battery materials.^24^ However, to date, there have been relatively few reports of techniques that can provide information at single particle level or possess the capability to distinguish variations in the electrochemical performance of individual active entities. The work presented herein demonstrates new capabilities of SECCM, which pave the way for the deep investigation of electrode reaction processes in energy conversion/storage technologies. In the future, we aim to visualize any minute influence of (nano)structure (e.g., crystallographic orientation) on redox activity and (de)intercalation kinetics through a combination of rational materials design and synthesis^25^ and SECCM. This will be achieved by investigating mono‐dispersed particles on TEM grids and then performing characterization by high‐resolution analytical TEM.

## Conflict of interest

The authors declare no conflict of interest.

## Supporting information

As a service to our authors and readers, this journal provides supporting information supplied by the authors. Such materials are peer reviewed and may be re‐organized for online delivery, but are not copy‐edited or typeset. Technical support issues arising from supporting information (other than missing files) should be addressed to the authors.

Supplementary

Supplementary
