# Supplementary material for: Correlative Electrochemical Microscopy of Li‐Ion (De)intercalation at a Series of Individual LiMn2O4 Particles
Source: Angew Chem Int Ed Engl. 2019 Feb 21;58(14):4606–11. doi: 10.1002/anie.201814505 (PMC6766856; doi:10.1002/anie.201814505)
Supplement: Supplementary file 1 — Supplementary [file ANIE-58-4606-s001.pdf]

Angewandte Chemie
Eine Zeitschrift der Gesellschaft Deutscher Chemiker

Supporting Information

Correlative Electrochemical Microscopy of Li-Ion (De)intercalation at a Series of Individual $LiMn_2O_4$ Particles

Binglin Tao, Lewis C. Yule, Enrico Daviddi, Cameron L. Bentley, and Patrick R. Unwin**

anie_201814505_sm_miscellaneous_information.pdf
anie_201814505_sm_movie_S1.avi

Table of Contents

Section 1	Experimental Section	3
Section 2	Supporting Figures	4-11
Section 3	Movie Caption	12
Section 4	Galvanostatic Charge/Discharge Process: Explanation and Calculations	12

Experimental Procedures

Chemical reagents and electrode preparation. Lithium manganese oxide ($LiMn_2O_4$, electrochemical grade) and lithium chloride (LiCl, ≥ 99 %) were purchased from Sigma-Aldrich, and used as received. Deionized water (resistivity ≥18 MΩ • cm) was produced by a Purite Integra HP system (U.K.). The glassy carbon (GC) plate was purchased from Alfa Aesar, and polished with 0.05 μm diamond suspension (Bueler, U.S.A.) prior to use. To prepare the working electrode, $LiMn_2O_4$ was sonicated in deionized water for 10 min, and then 1 μL of the suspension was drop casted onto the GC substrate. The silver/silver chloride (Ag/AgCl) quasi reference counter electrode (QRCE) was prepared by anodic polarization of an Ag wire (0.125 mm diameter, Goodfellow, 99.99 %) in saturated KCl solution. The QRCE potential was calibrated against a commercial saturated calomel electrode (SCE) in 1 M LiCl solution before and after each experiment, and was found to possess a stable reference potential of ± 0.005 V *vs.* SCE.

The composite battery macro-electrode (working electrode) was prepared as follows: active material (85 wt%), acetylene black (10 wt%)and polytetrafluoroethylene (PTFE, 5 wt%) aqueous solution were mixed together, followed by sonication treatment for 20 min and drying for 1 h. Approximately 2.8 mg of the mixture was impregnated into a stainless titanium grid with area of 1 cm^2 and then pressed under 5 MPa pressure to fabricate the electrode. The working electrode was soaked with electrolyte solution overnight, prior to use to ensure completing wetting.[1]

Instrumentation. Single channel nanopipettes with diameter of 500 nm and 8 μm were prepared using a CO_2 laser puller (P-2000, Sutter Instruments, U.S.A.).The former were pulled from glass capillaries (GC120F-10 1.2OD×0.69×100 mm, Harvard Apparatus, U.S.A.) with a two-step protocol. For the first step, the parameters were heat 350, filament 3, velocity 40 and delay 220. For the second step, the parameters were heat 350, filament 3, velocity 40, delay 180 and pull 100. The dimensions of the nanopipette orifice were measured using scanning electron microscopy (SEM) on a Zeiss Supra 55VP system, which was operated at an accelerating voltage of 2 kV. Typically, the diameter of the nanopipette probe was 500-600 nm. The latter were pulled from glass capillaries (GC100F-7.5 1.0OD×0.58ID, Harvard Apparatus, U.S.A.), and a one-step protocol was exploited. The parameters were heat 350, filament 3, velocity 40 and delay 220. Single channel nanopipettes with diameter of 50 μm were pulled from glass capillaries (GC100F-7.5 1.0OD×0.58ID, Harvard Apparatus, U.S.A.) using a PC-10 puller (Narishige Group, Japan) with a two-step protocol. For the first step, the parameters were heater 70, weight 3, and slider 8. For the second step, the parameters were heater 55, weight 3, and slider 4. After pulling, the nanopipette probes were filled with a 1 M LiCl solution using a MicroFil syringe (World Precision Instrument Inc., U.S.A.), and a QRCE was inserted from the back.

Macroscale 'bulk' electrochemical experiments were carried out in the 3-electrode format (graphite rod and commercial Ag/AgCl reference electrodes for counter and reference electrodes, respectively) on a CHI 660D electrochemical work station. All experiments in the SECCM configuration were carried out on a home-built scanning electrochemical probe microscopy (SEPM) platform.[2] As shown schematically in Figure S1, in this configuration, the prepared nanopipette probe and substrate of interest were mounted on a z-(P-753.3CD, Physik Instrumente, Germany) and *xy*-(P-622.2CD, Physik Instrumente) piezoelectric positioners, respectively, which were controlled by amplifier modules (E-665 and E-500, respectively). Coarse and fine control of the nanopipette probe with respect to the substrate surface was achieved with micropositioners and piezoelectric positioners (detailed above), respectively. The SECCM cell and all piezoelectric positioners were placed in an aluminum Faraday cage, which was installed on an optical table (RS2000, Newport, U.S.A.) with automatic leveling isolators (Newport, S-2000A-423.5).During experiments, the current was measured every 4 μs, and averaged 513 times to give a data acquisition rate of 2.052 ms per point. This current signal was typically filtered using an 8th order low-pass filter at a time constant of 2 ms. Data acquisition and fine control of the whole system was achieved on a Field Programmable Gate Array (FPGA) board (PCIe-7852R) controlled by a Labview 2016 (National Instruments, U.S.A.) interface running the Warwick Electrochemical Scanning Probe Microscopy (WEC-SPM,www.warwick.ac.uk/electrochemistry) software.

Scanning protocols. Electrochemical measurements on the substrate (working electrode) were performed using three different 'scan hopping' modes, including 'scan hopping with cyclic voltammetry', 'scan hopping with current-time' and 'scan hopping with galvanostatic charge-discharge'. Taking 'scan hopping with cyclic voltammetry' as an example (see below, Figure S2), the nanopipette (meniscus cell) was approached to the surface of interest at an approach rate of 3 μm/s, during which the current was monitored constantly. Upon landing, *i.e.*, when an electrochemical cell was formed between the nanopipette and sample surface through the meniscus, the approach was stopped immediately after detecting a threshold current of 1.2 pA (set to be slightly larger than the noise level). It should be noted that the nanopipette probe itself did not physically contact with the substrate. After detecting the surface, a local cyclic voltammetric experiment was performed, following which the nanopipette was retracted 5 μm from the surface. The nanopipette was subsequently moved to next predefined pixel, located 1.5 or 2 μm from the previous point, set by the predefined 'hopping distance'. At each pixel, the vertical extension of the nanopipette (z-coordinate) and electrochemical signals (potential, *E* and current, *i*) were recorded synchronously, effectively building up topographical and voltammetric 'activity' maps, respectively. The working principles of 'scan hopping with current-time' and 'scan hopping with galvanostatic charge-discharge' were very similar, except chronoamperometry or galvanostatic charge-discharge (chronopotentiometry) was carried out as substitutes for cyclic voltammetry at each pixel. After the SECCM scanning experiments, the probed (working electrode) area was determined by imaging the droplet footprint left behind at each pixel with SEM (Zeiss Gemini 500).

Data processing. After collection, the raw data were processed with Matlab R2015b software package. Sample tilt was removed using SPIP v. 6.0.14 software package. Data plotting was performed using Matlab R2015b and OriginPro 2016 software packages. It should be noted that cyclic voltammograms presented in the main text have been smoothed by averaging with 10 adjacent points.

Supporting Figures

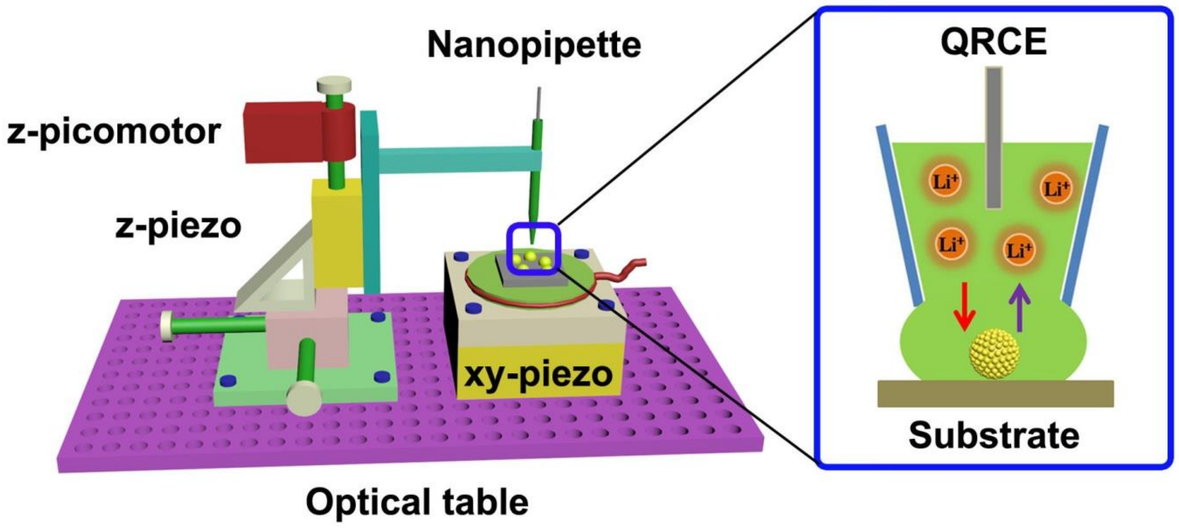

Figure S1. Labeled schematic of SECCM setup employed herein. The nanopipette probe is fixed to a *z*-piezoelectric positioner (fine movement, labelled z-piezo in the image), which is mounted on a z-picomotor and *xy* micropositioners for coarse movement. The sample is mounted on a *xy*-piezoelectric positioner (labelled xy-piezo in the image) for fine control of lateral position. On the right is an enlarged diagram of the probe-particle-support interface during a single 'hop' of a scanning experiment. Electrochemical experiments are performed by applying a potential at the QRCE in the nanopipette barrel (with respect to ground), while measuring the current at the substrate surface (at ground).

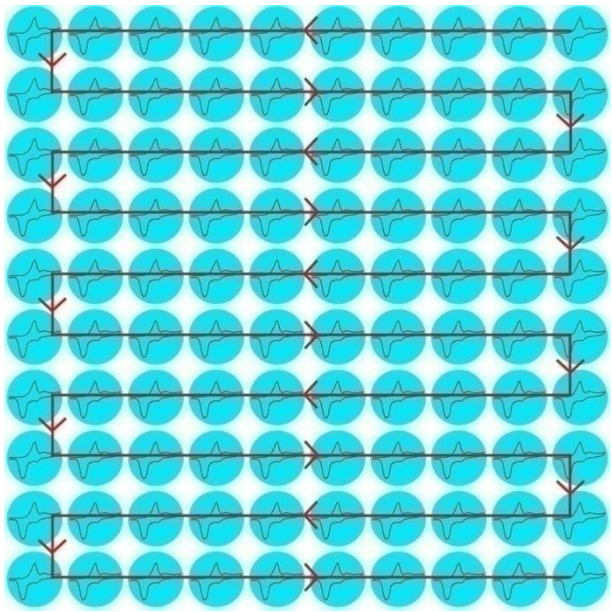

Figure S2. Schematic diagram illustrating the scanning route (red arrows) during a 'scan hopping with cyclic voltammetry' experiment. An independent cyclic voltammetric experiment is carried out at each and every point, building up a pixel-resolved activity map of the substrate. The blue circles represent the droplet 'footprint' (*i.e.*, probed area of the meniscus cell).

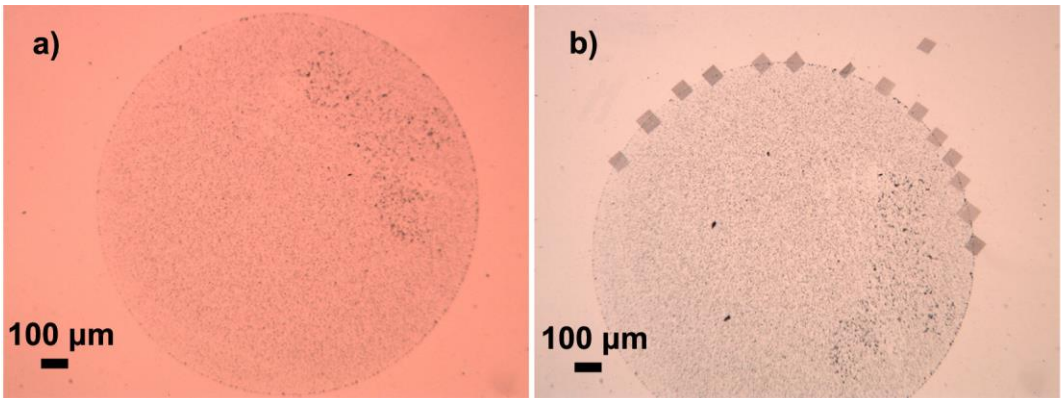

Figure S3. Optical micrograph of a $LiMn_2O_4$/GC electrode, which was prepared by drop-casting method. **a)** and **b)** were taken before and after SECCM scanning, respectively. Note that there is a rotation of b, with respect to a by about 40-50°. The 'squares' in b are the arrays of dots of successful SECCM experiments, most of which were carried out at the border between the drop-cast region and GC substrate. One experiment was carried out on GC alone. One scan is rectangular rather than square, indicating an incomplete scan (This scan was stopped manually). Overall, Figure S3b, indicates the high success rate of SECCM scanning and the ability to position the tip in a desired area.

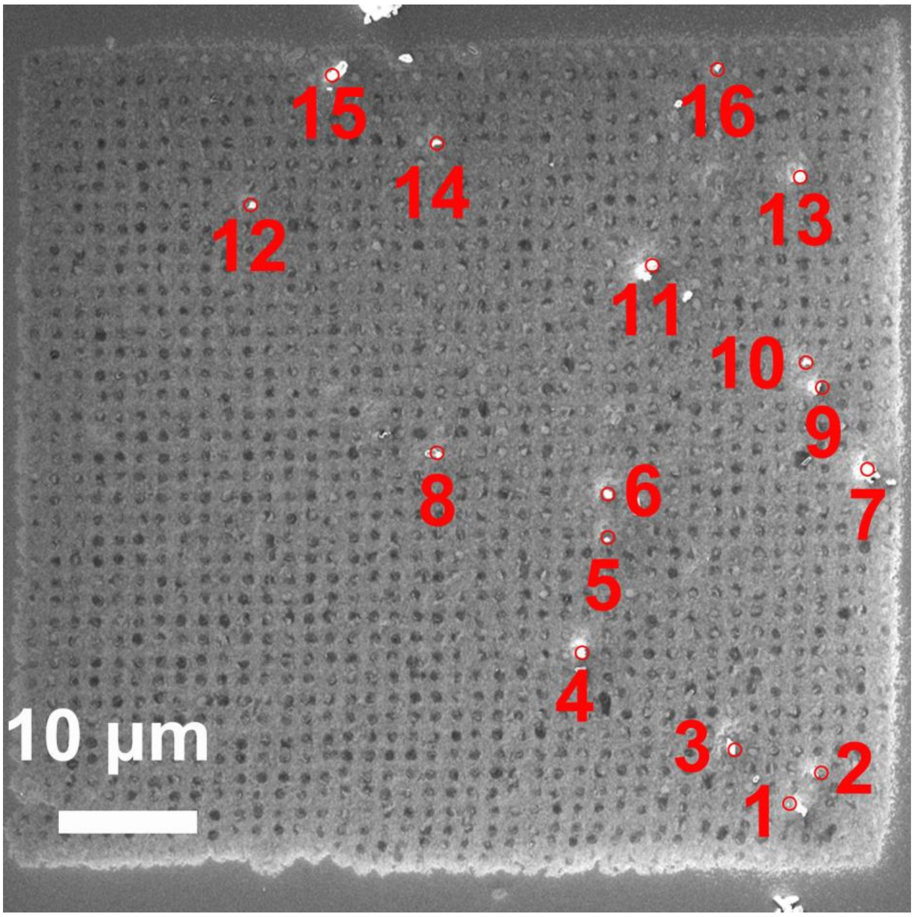

Figure S4. SEM image of the scanning area of the cyclic voltammetry measurements, as shown in **Figure 2** of the main text. The red numbers correspond to the pixel-resolved CVs and corresponding SEM images of the $LiMn_2O_4$ particles, shown in **Figure S6** and **S7**, respectively.

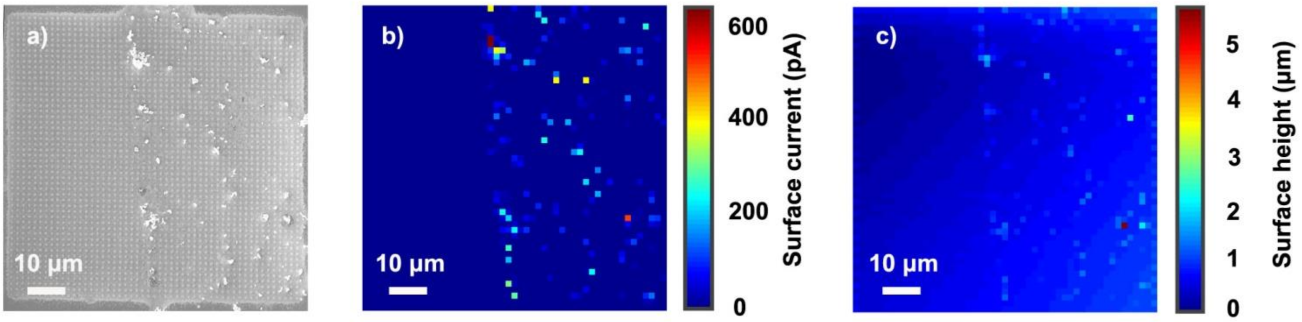

Figure S5. Chronoamperometric (*I-t*) SECCM measurements performed at individual and aggregated $LiMn_2O_4$ particles supported on GC at a fixed potential of +1.0 V *vs.* Ag/AgCl QRCE and pulse tine lasted for 1s. **a)** SEM image, **b)** surface current map at 0.5s and **c)** topography of the corresponding scanning area..

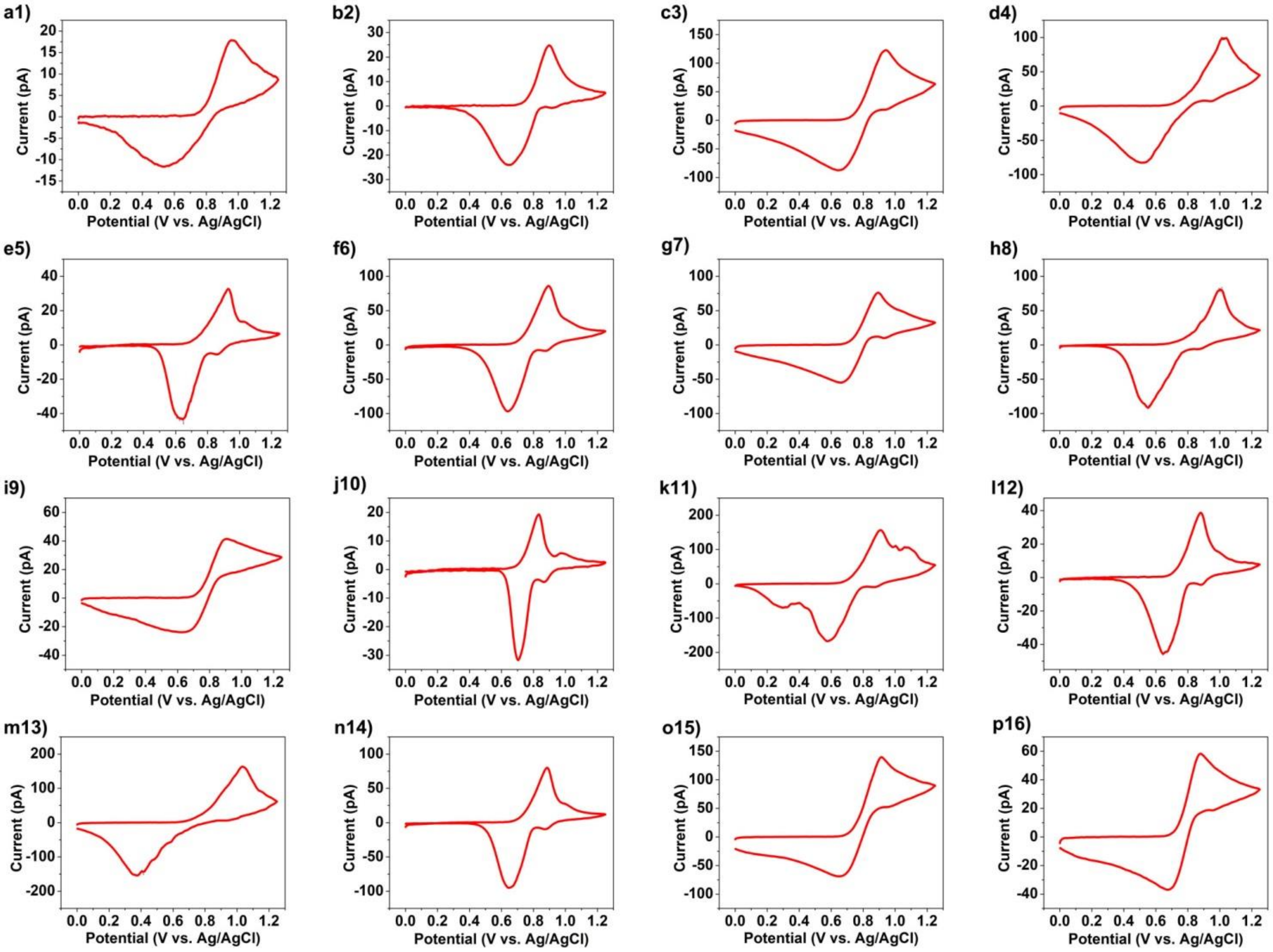

Figure S6. 16 independent cyclic voltammograms (CVs) obtained at the individual $LiMn_2O_4$ particles labeled in **Figure S4**, with corresponding high resolution SEM images shown in **Figure S7**. Note that these particles (or particle agglomerates) are not necessarily fully encapsulated by the meniscus (droplet) cell during scanning. These experiments were performed in 1 M LiCl, with a 500 nm diameter probe and at a scan rate (*v*) of 1 V/s.

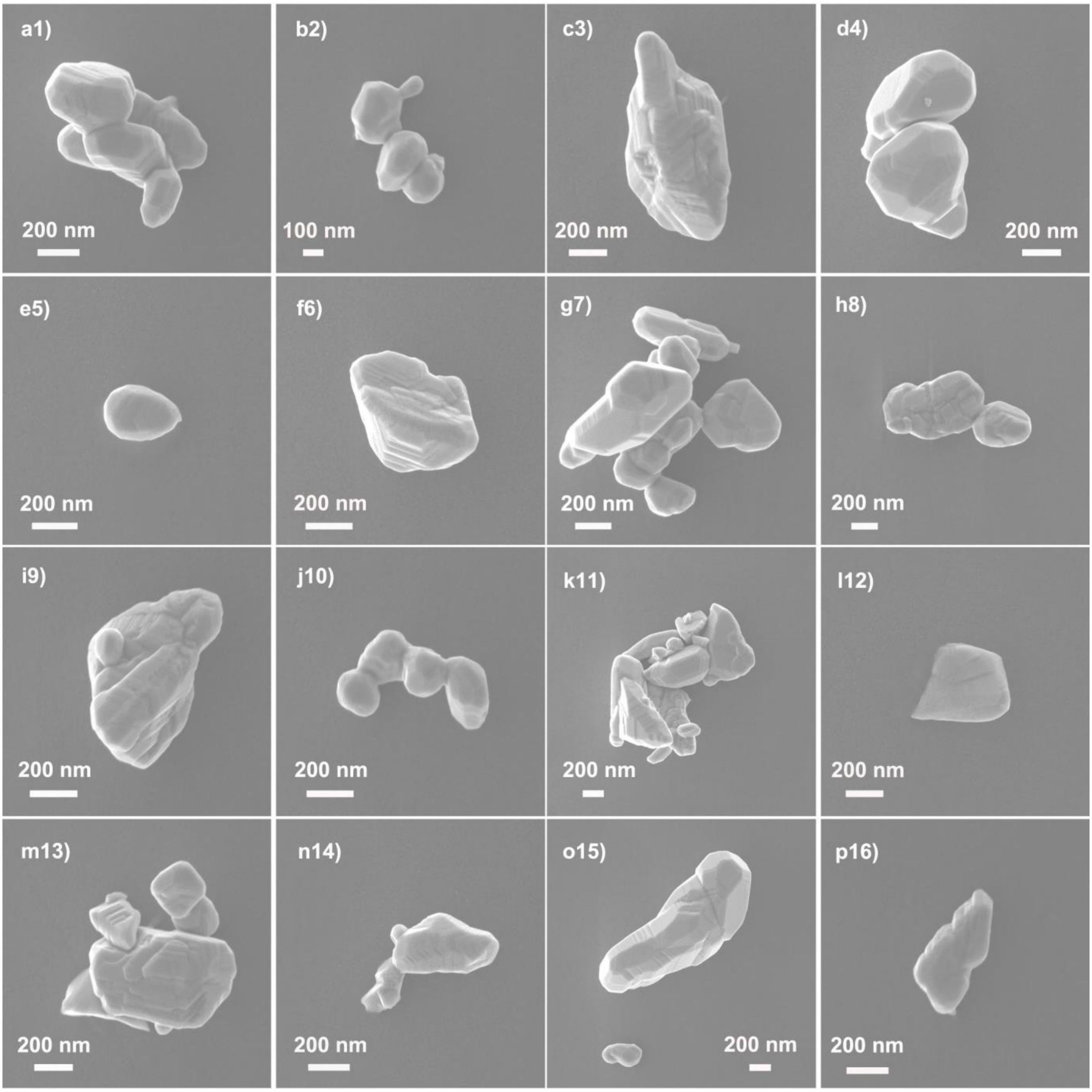

Figure S7. High resolution SEM images of individual $LiMn_2O_4$ particles probed with CV-SECCM, as shown in **Figure S4** and **S6**.

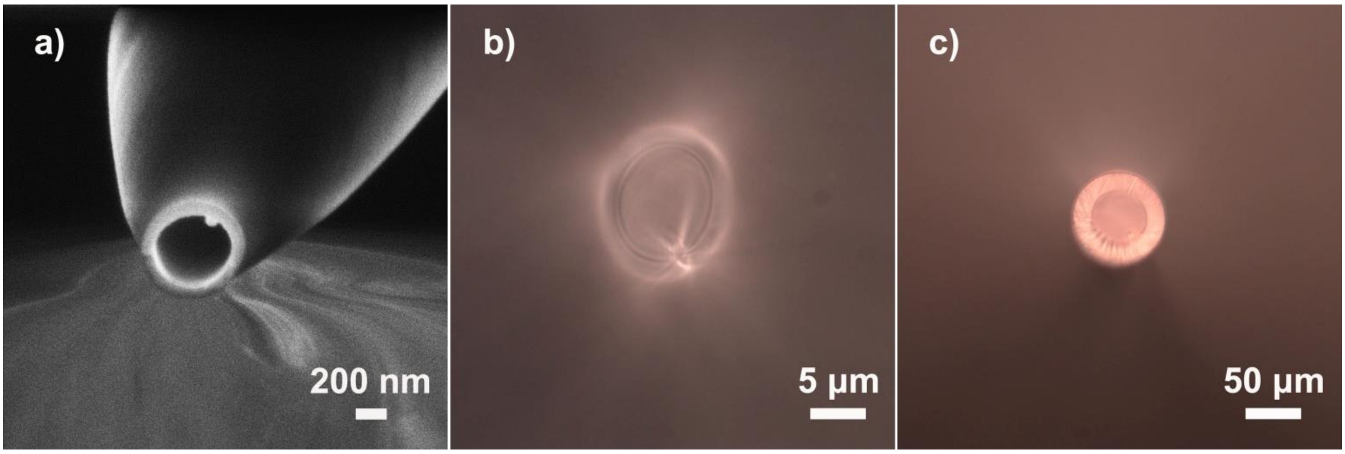

Figure S8. a) SEM image of a representative nanopipette probe. The inner diameter of this tip is about 500 nm, and the inside filament could be observed clearly, indicating the robustness of the two-step pulling protocol. **b)** and **c)** Optical microscopic images of representative micropipette probes with diameters of 8 and 50 µm, respectively.

Table S1. Electrochemical characteristics of the 8 $LiMn_2O_4$ particles shown in the main text **Figure 3** and SI, **Figure S9**, derived from single-particle cyclic voltammetry measurements.

Particle	Oxidation peak					Reduction peak									
	E_{p1}/V	E_{p2}/V	i_{p1}/pA	i_{p2}/pA	Q/pC	E_{p1}/V	E_{p2}/V	i_{p1}/pA	i_{p2}/pA	Q/pC	ΔE_{p1} /V	ΔE_{p2} /V	Reduction to oxidation charge ratio/%	Volume[a] /$cm^3 \times 10^{-14}$	Capacity usage/%[b]
a	0.93	1.04	33.00	13.78	6.39	0.64	0.87	43.27	5.41	8.73	0.29	0.17	136.62	1.01	29.1
b	0.88	1.00	38.50	15.25	7.63	0.66	0.89	44.59	4.62	10.00	0.22	0.11	131.06	2.09	16.7
c	0.83	0.98	29.24	11.48	5.14	0.76	0.82	59.74	13.00	6.13	0.07	0.16	119.26	2.28	10.3
d	0.86	0.98	46.92	22.2	9.67	0.71	0.87	62.12	15.04	11.61	0.15	0.11	120.06	2.80	15.8
e	0.87	1.00	50.84	23.26	10.16	0.71	0.89	57.06	9.81	12.92	0.16	0.11	127.17	3.77	12.3
f	0.85	0.99	39.2	14.43	6.79	0.75	0.88	75.56	13.43	7.81	0.10	0.11	115.02	2.41	12.9
g	1.08	0.17	73.74	46.86	15.82	0.47	0.83	86.83	10.36	24.1	0.61	0.34	152.34	4.50	16.1
h	1.04	-	24.94	-	5.63	0.73	0.95	35.62	7.80	6.96	0.31	-	123.62	1.22	21.1

E_p and i_p correspond to peak potential and current, respectively. The subscripts ‘1’ and ‘2’ denote the first and second pair of peaks, respectively. Q is the charge, calculated by integrating the peaks. ΔE_p is the peak-to-peak separation. Reduction to oxidation charge ratio was calculated as follows: $Q_{reduction}/Q_{oxidation} \times 100\%$. [a] The volume of the particle was estimated based on the height (SECCM topography), length and width (SEM image). [b] Capacity usage = real capacity/theoretical capacity × 100%. The real capacity is calculated from the CV curve, while the theoretical capacity is calculated based on the volume.

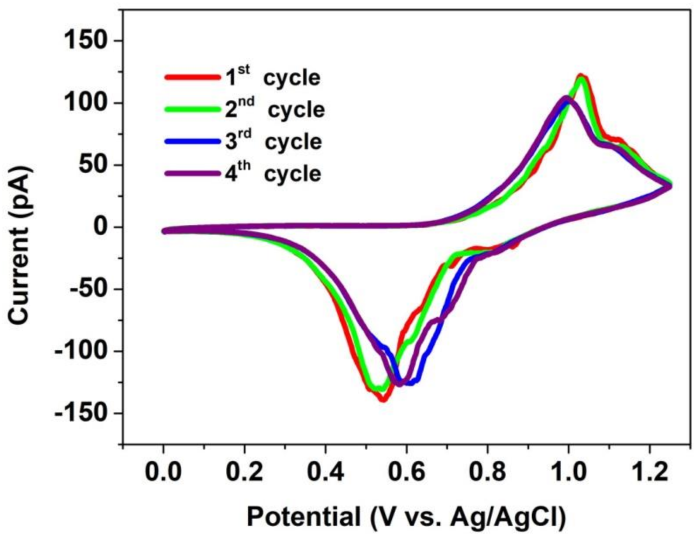

Figure S9. First 4 CVs obtained at individual $LiMn_2O_4$ particles. The experiment was performed at a scan rate (v) of 1 V/s. It should be noted that the peak position moved during the 3rd and 4th cycle, indicating the (de)intercalation reaction became much more facile.

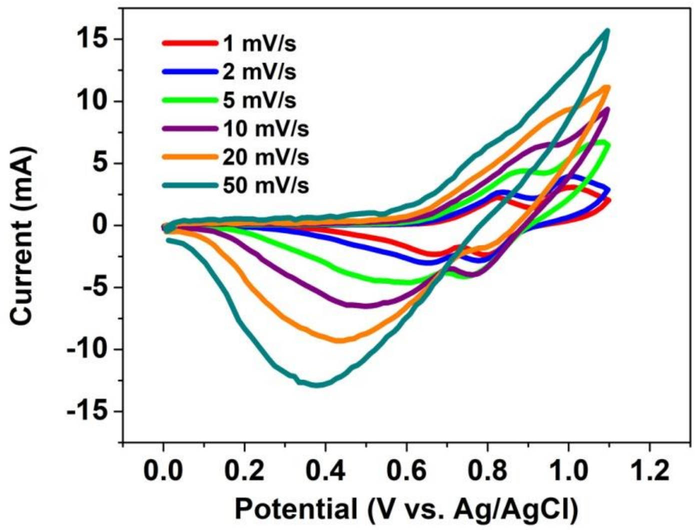

Figure S10. Macroscopic CVs obtained from a composite $LiMn_2O_4$ electrode, at scan rates ranging from 1 to 50 mV/s. Two pairs of (de)intercalation peaks can be observed only at slow scan rates (less than 5 mV/s). This limitation in (de)intercalation kinetics is attributable to sluggish charge transfer within the complex composite electrode matrix, as addressed in the main text.

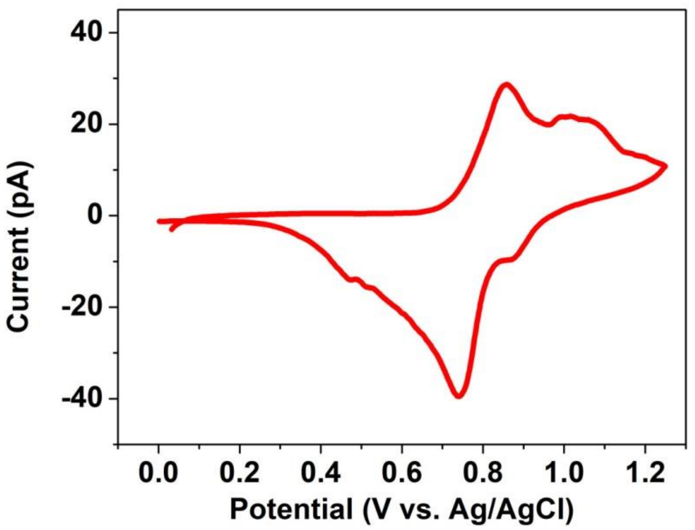

Figure S11. Averaged CV profile from 8 independent particles in Figure 3.

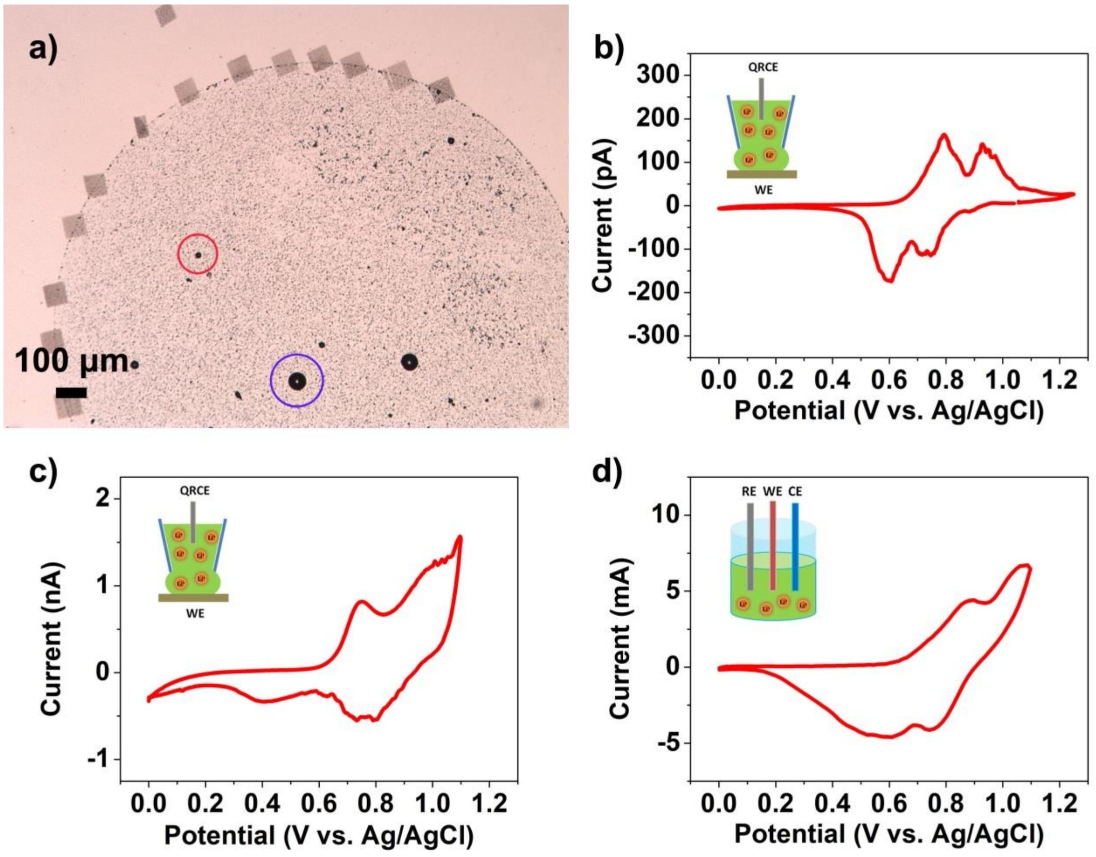

Figure S12. a) Optical micrograph of a $LiMn_2O_4$/GC electrode. The red and blue circles represent the droplet 'footprints' (*i.e.*, probed area of the meniscus cell) of measurements performed with probes of diameter 8 and 50 µm, respectively. **b)** Experiment performed with a tip of 8 µm diameter, at v = 20 mV/s. The CV profile shows two pairs of symmetrical peaks, analogous to that obtained in bulk with the composite electrode at v = 1 mV/s (shown in **Figure S10**). **c)** Experiment performed with a tip of 50 µm diameter, at v = 5 mV/s. **d)** Bulk measurement performed with a composite electrode at v = 5 mV/s (extracted from **Figure S10**). The results obtained from SECCM at the microscale, b) and c), are consistent with the bulk measurements with the composite electrode, d), indicating that the diversity of responses obtained at the single particle level (*e.g.*, Figure 3 in the main text) must arise from intrinsic differences between the $LiMn_2O_4$ particles (*i.e.*, kinetic effects), rather than contamination or an artefact from the SECCM configuration.

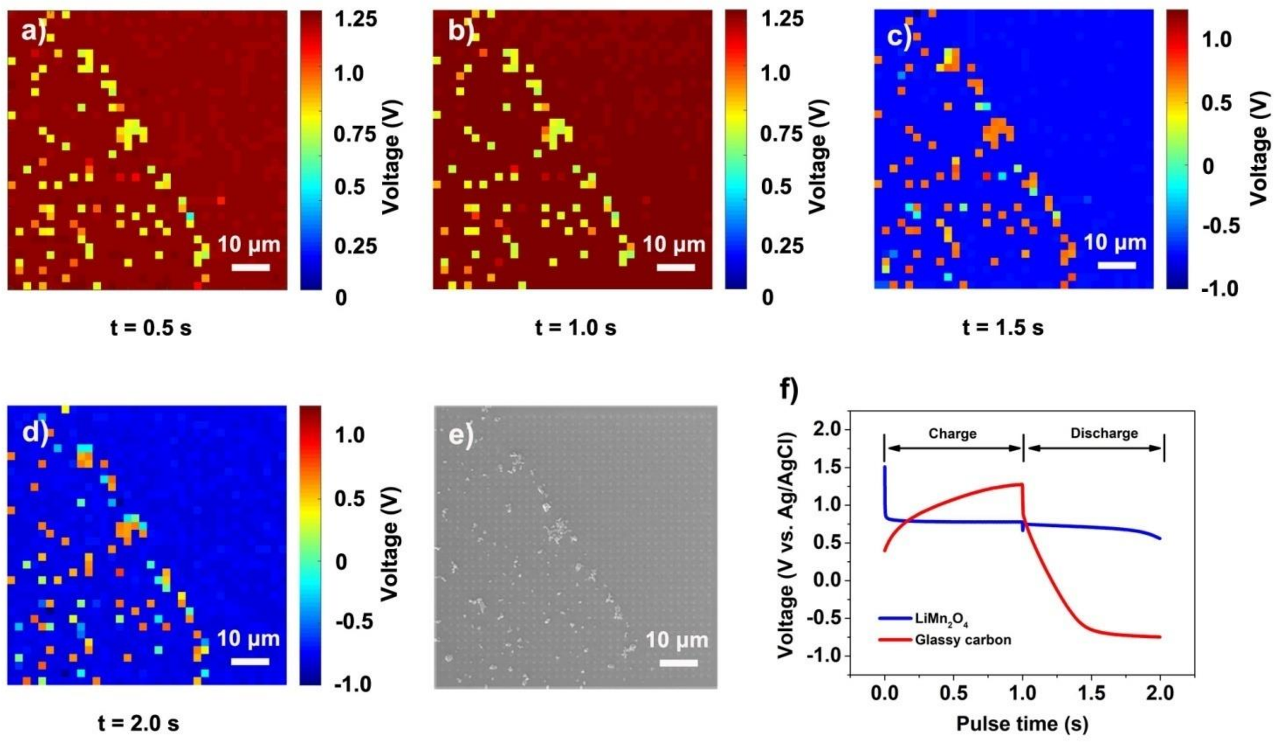

Figure S13. Galvanostatic charge-discharge measurements of individual and aggregated $LiMn_2O_4$ particles supported on GC. **a), b)**, **c)** and **d)** spatially resolved potential images at different times of 0.5, 1.0, 1.5, and 2.0 s during galvanostatic charging-discharge, respectively. **e)** SEM image of the corresponding scanning area. **f)** Potential-time characteristics of GC (red) and $LiMn_2O_4$ particle (blue). The galvanostatic charge/discharge measurements were obtained by local ensemble measurements with SECCM, with a 500 nm diameter probe filled with 1 M LiCl.

Movie Caption

Movie S1.Spatially resolved CV-SECCM movie obtained from Li^+ intercalation/deintercalation on $LiMn_2O_4$ particles supported on GC. This experiment was performed in 1 M LiCl, with a 500 nm diameter probe and at a scan rate (*v*) of 1 V/s.

Galvanostatic Charge/Discharge Process: Explanation and Calculations

Galvanostatic charge/discharge measurements performed in SECCM differ from the convention commonly applied for bulk electrochemical experiments.[3] In this work, if we assume that the $LiMn_2O_4$ particle is roughly spherical with 'average' diameter of 0.5 μm, then the volume of a typical particle would be:

V=4/3*πr^3= 4÷3×3.14× $(0.25)^3$ $μm^3$ = 0.065 $μm^3$ =0.65 × 10^{-13} cm^3

Note that the actual volume of each particle can be estimated based on the height (estimated from z-height topography), width and length (estimated from SEM image) by assuming the particle is an ellipsoid (V = 4/3πabc), as shown in **Table 1**, main text. Considering the capacity and density are 148 mAh/g and 4.1 g/cm^3, respectively,[4] the theoretical volumetric capacity (C_v) of these $LiMn_2O_4$ particles is *ca.* 600 mAh/cm^3. Thus, if we want to fully charge/discharge each individual particle, the total charges (Q_t) we that we would need to apply are:

Q_t= 0.65 × 10^{-13} cm^3× 606.8 mA h/cm^3 = 0.395× 10^{-10} mA h = 0.0395 pA h = 142.2 pA s

In the SECCM configuration, as we only apply a small current and short contact time with the pipette (Q = 5 pA s), the particle are only charged/discharged a small fraction of the total (volumetric) capacity. As a result, a relatively constant *E-t* profile is observed, centred around a potential of *ca.* 0.75 V *vs.* Ag/AgCl. For comparison, in macroscale tests, C-rates ranging from 0.1 C to 10 C (*i.e.*, 10 hours to 0.1 hour charge/discharge time, respectively) are usually performed to fully charge/discharge the battery electrode.[5]

References

[1] J.Y. Luo, W.J. Cui, P. He, Y.Y. Xia, *Nature chemistry* **2010**, *2*, 760-765.
[2] a) M. Kang, P. Wilson, L. Meng, D. Perry, A. Basile, P. R. Unwin, *Chem. Commun.* **2018**, *54*, 3053-3056; b) A. Page, M. Kang, A. Armitstead, D. Perry, P. R. Unwin, *Anal. Chem.* **2017**, *89*, 3021-3028.
[3] I. Izanzar, M. Dahbi, M. Kiso, S. Doubaji, S. Komaba, I. Saadoune, *Carbon* **2018**, *137*, 165-173.
[4] X. Li, S. Guo, H. Deng, K. Jiang, Y. Qiao, M. Ishida, H. Zhou, *Journal of Materials Chemistry A* **2018**, *6*, 15517-15522.
[5] a) G. Wang, L. Fu, N. Zhao, L. Yang, Y. Wu, H. Wu, *Angew. Chem.* **2007**, *119*, 299-301; b) A. Tron, Y. D. Park, J. Mun, *J. Power Sources* **2016**, *325*, 360-364.
